# Characterization and comparative analysis of antibiotic resistance plasmids isolated from a wastewater treatment plant

**DOI:** 10.3389/fmicb.2014.00558

**Published:** 2014-10-28

**Authors:** Teddie O. Rahube, Laia S. Viana, Günther Koraimann, Christopher K. Yost

**Affiliations:** ^1^Department of Biology, University of ReginaRegina, SK, Canada; ^2^Department of Biology and Biotechnological Sciences, Botswana International University of Science and TechnologyPalapye, Botswana; ^3^Institute of Molecular Biosciences, University of GrazGraz, Austria

**Keywords:** antibiotic resistance genes, plasmids, conjugation, genetic, wastewater, mobile genetic elements

## Abstract

A wastewater treatment plant (WWTP) is an environment high in nutrient concentration with diverse bacterial populations and can provide an ideal environment for the proliferation of mobile elements such as plasmids. WWTPs have also been identified as reservoirs for antibiotic resistance genes that are associated with human pathogens. The objectives of this study were to isolate and characterize self-transmissible or mobilizable resistance plasmids associated with effluent from WWTP. An enrichment culture approach designed to capture plasmids conferring resistance to high concentrations of erythromycin was used to capture plasmids from an urban WWTP servicing a population of ca. 210,000. DNA sequencing of the plasmids revealed diversity of plasmids represented by incompatibility groups *Inc*U, *col*-E, *Inc*FII and *Inc*P-1β. Genes coding resistance to clinically relevant antibiotics (macrolide, tetracycline, beta-lactam, trimethoprim, chloramphenicol, sulphonamide), quaternary ammonium compounds and heavy metals were co-located on these plasmids, often within transposable and integrative mobile elements. Several of the plasmids were self-transmissible or mobilizable and could be maintained in the absence of antibiotic selection. The *Inc*FII plasmid pEFC36a showed the highest degree of sequence identity to plasmid R1 which has been isolated in England more than 50 years ago from a patient suffering from a *Salmonella* infection. Functional conservation of key regulatory features of this F-like conjugation module were demonstrated by the finding that the conjugation frequency of pEFC36a could be stimulated by the positive regulator of plasmid R1 DNA transfer genes, TraJ.

## Introduction

Wastewater treatment plants (WWTPs) have been recognized as reservoirs for antibiotic resistance genes that are associated with human pathogens (Schluter et al., 2007; Szczepanowski et al., 2005; Rahube and Yost, 2010; LaPara and Burch, 2011; LaPara et al., 2011; Rizzo et al., 2013). This is mainly because WWTPs receive human fecal wastes from households containing excreted gut bacteria including antibiotic resistant bacteria (ARB). The gastrointestinal microbiota is a primary source of resistant bacteria shed with human waste (Sommer et al., 2009). These ARB and antibiotic residues are excreted in urine and feces, and are ultimately transported to WWTPs via domestic sewer lines (Hirsch et al., 1999). Antibiotic residues that are consistently detected in municipal WWTPs include macrolides (erythromycin, clarithromycin, roxithromycin), lincosamide (lincomycin), tetracyclines (tetracycline, oxytetracycline), sulphonamides (sulfamethazine, sulfamethoxazole), and fluoroquinolones (ciprofloxacin, norfloxacin) (Giger et al., 2003; Karthikeyan and Meyer, 2006).

The importance to public health of investigating the dissemination of the antibiotic resistance genes (“resistome”) and mobile genetic elements that harbor these resistance genes (“mobilome”) in non-clinical environments has been highlighted by several recent review articles (Ashbolt et al., 2013; Gaze et al., 2013; Gillings, 2013; Perry and Wright, 2013; Purden, 2013). The need to increase our understanding of the environmental dimension of antibiotic resistance is emphasized by the continued rise in infections caused by multiple antibiotic resistant pathogenic bacteria. Plasmids that carry antibiotic resistance genes may represent a particular public health risk because antibiotic resistance in infectious bacteria is often associated with a plasmid (Palmer et al., 2010). Furthermore, these plasmids are frequently mobilizable and prone to accumulating mobile antibiotic resistance gene cassettes. Plasmid mobility is important in the evolution and dissemination of multiple antibiotic resistance in bacteria found in the different environments (Smillie et al., 2010). This paper describes the characterization of previously non-described multiple resistance plasmids isolated from the Regina (Saskatchewan, Canada) WWTP influent and effluent water isolated using an *Escherichia coli* strain to capture plasmids that conferred resistance to high concentrations of erythromycin.

## Materials and methods

### Description of the regina wastewater treatment plant

The city of Regina (Saskatchewan, Canada) WWTP services a population of 210,556 and processes domestic, hospital, and pre-treated oil refinery waste-water. Biological treatment occurs with aerated lagoons and effluent receives a final treatment of UV disinfection. The effluent is discharged into Wascana Creek. Waste-water treatment plant samples included water from both the primary influent as well as the released treated. Samples were collected by WWTP staff and transported back to the lab in coolers.

### Plasmid isolation technique and DNA sequencing

Plasmids were isolated from the influent and effluent water samples using an enrichment culture technique previously described by Rahube and Yost (2012). Briefly, following water collection and transport to the laboratory, samples were vortexed vigorously and solid particles were allowed to settle for 30–45 min before the liquid was vacuum filtered through a 0.45 μm membrane filter. The bacteria trapped on the filter were re-suspended in phosphate buffered saline and plated onto LB agar supplemented with 400 μg mL^−1^ of erythromycin. Plasmid DNA was isolated from the pooled bacterial culture using a NucleoBond® Xtra Midi prep kit (Macherey Nagel, Duren, Germany) according to the manufacturer's instructions. The purified plasmid DNA was used to transform high efficiency *E. coli* DH10β competent cells according to manufacturer's specifications (New England BioLabs, Inc., Canada). The transformation mixture was plated on LB containing 400 μg mL^−1^ of erythromycin. Resultant erythromycin resistant colonies were screened for resistance to multiple classes of antibiotics by transferring colonies to LB agar plates that were individually supplemented with various antibiotic classes. The specific antibiotics used to test for multiple resistance included: ampicillin (100 μg mL^−1^), tetracycline (10 μg mL^−1^), gentamicin (15 μg mL^−1^), streptomycin (100 μg mL^−1^), neomycin (20 μg mL^−1^), chloramphenicol (25 μg mL^−1^), and rifampicin (30 μg mL^−1^). Plasmid size was measured using an Eckhardt protocol for plasmid visualization following direct cell lysis in agarose gels (Hynes et al., 1985). Purified DNA from four distinct plasmids, based on antibiotic resistance profiles and differences in plasmid size were sent for next generation 454 DNA sequencing at the DNA Core Facility at the Ontario Agency for Health Protection and Promotion (Toronto, ON, Canada). The plasmid DNA was sequenced using a Roche GS-FLX sequencer yielding an average of 25X sequence coverage.

### Gene annotations and bioinformatics analyses

The sequence data obtained were imported and assembled with Sequencher computer software (GeneCodes® Corporation, Ann, Arbor, Michigan). A primer walking and polymerase chain reaction (PCR) cloning strategy was used to close the gaps between assembled contigs. Subsequent DNA sequencing of the PCR amplicons was performed to confirm correct completed plasmid sequence assembly. The complete consensus sequences were arranged so that the sequence begins with the predicted replication genes at the start of the plasmid sequence. The sequences were imported into the Rapid Annotation Subsystem Technology (RAST) server for gene predictions (Aziz et al., 2008). The putative conserved domain analysis of translated open reading frame (ORF) protein sequences were performed using the PSI-BLAST on the National Center for Biotechnology Information server (http://www.ncbi.nlm.nih.gov/blast/Blast.cgi). Genbank files of annotated plasmid sequences were imported from the RAST server into Vector NTI 10.3.0 (Invitrogen Corporation, Carlsbad, CA) for generation of visual maps. The annotated nucleotide sequences of the plasmids pTOR_01, pTOR_02, pEFC36a and pRWC72a are available in the Genbank database under accession numbers JX843237, JX843238, JX486126, and JX486125, respectively.

Comparative genomic analyses of the plasmid sequences were completed using progressive mauve multiple genome alignment software version 2.0 (http://gel.ahabs.wisc.edu/mauve/) (Darling et al., 2010). Phylogenetic guide trees were generated using clustalW2 sequence alignment tool on the European Bioinformatics Institute (EBI) server (http://www.ebi.ac.uk/Tools/msa/clustalw2/). The plasmid sequences used for comparative genomic analysis were imported from the Genbank database into Vector NTI and were adjusted such that they all start with the replication gene at position one.

### Functional analysis of plasmid mobility and stability

Plasmid mobility was determined by conjugation experiments on LB agar and Vincent's minimal medium (VMM) as described previously by Rahube and Yost (2012). The donors strains were commercially available *E. coli* cloning strains DH5α, *E. coli* DH10β (Invitrogen Corporation, Carlsbad, CA) and *E. coli* S17-1 containing the plasmid RP-4 *tra* genes integrated within the chromosome (Simon et al., 1983) and *Pantoea agglomerans* 5565 (Nadarasah and Stavrinides, 2014). The recipient strains included kanamycin resistant derivatives of *E. coli* DH5α, *P. ananatis* BRT175 and rifampicin resistant *P. agglomerans* 5565. The *Pantoea* species were kindly supplied by Dr. John Stavrinides, University of Regina and were selected as environmental and opportunistic pathogens that are also members of the γ-proteobacteria. *P. agglomerans* 5565 was isolated as an endophyte from soybean while *P. ananatis* BRT175 was isolated from as strawberry epiphyte (Nadarasah and Stavrinides, 2014). Transconjugants in the conjugation experiments were confirmed for plasmid carriage by PCR amplification of target genes coding for plasmid replication and for macrolide resistance. Conjugation transfer frequencies were calculated as the number of transconjugants per donor cell (Soda et al., 2008).

We also investigated the activation of pEFC36a conjugation by the TraJ regulator found on the conjugative *Inc*FII resistance plasmid R1. R1 was originally isolated in 1962 from a patient suffering from a *Salmonella* infection in Brighton, England (Datta and Kontomichalou, 1965; Meynell and Datta, 1967). We first constructed a derivative of pEFC36a where the complete region conferring erythromycin resistance and predicted heavy metal resistance was replaced by a kanamycin resistance cassette amplified from the expression vector pET28a (Novagen). Gene replacement was performed using *E. coli* DY330 (Yu et al., 2000). Homologous regions corresponding to the *merR* and *catA* genes of pEFC36a were fused to the Kan^R^ cassette by megaprimer PCR (primer sequences are provided in the Supplemental Information), the resulting DNA fragment was gel purified and transformed into electro-competent *E. coli* DY330 containing pEFC36a. Kan^R^ clones were selected and characterized by PCR and restriction enzyme digests of isolated plasmid DNA. The resulting plasmid with the Kan^R^ cassette inserted between nt 59,980 and 85,114 of the original pEFC36a plasmid was named pEFC36a::Kan. This plasmid was assayed for its conjugation frequency in the presence of a second compatible plasmid, pJR1, expressing R1 *traJ*, or the control plasmid pGZSNO2, as described previously (Wagner et al., 2013).

*E. coli* DH5α and *P. agglomerans* 5565, were used as plasmid hosts for the assessment of plasmid stability. A single colony of the bacterium, containing the plasmid under study, was inoculated in LB broth without antibiotic selection and grown overnight at 37°C with agitation. One hundred microliter of the bacterial cells were aseptically transferred into fresh LB broth without antibiotic selection on consecutive days for up to 26 day. Total viable cells and plasmid containing cells were enumerated using serial dilutions and plating on both LB agar and LB agar with the appropriate antibiotic selection. Enumeration of total viable cells and plasmid containing cells was performed at days 2, 4, 8, 16, 24, and 26.

## Results

### Genetic characterization of the plasmids conferring erythromycin resistance isolated from the WWTP

**Figure 1:**
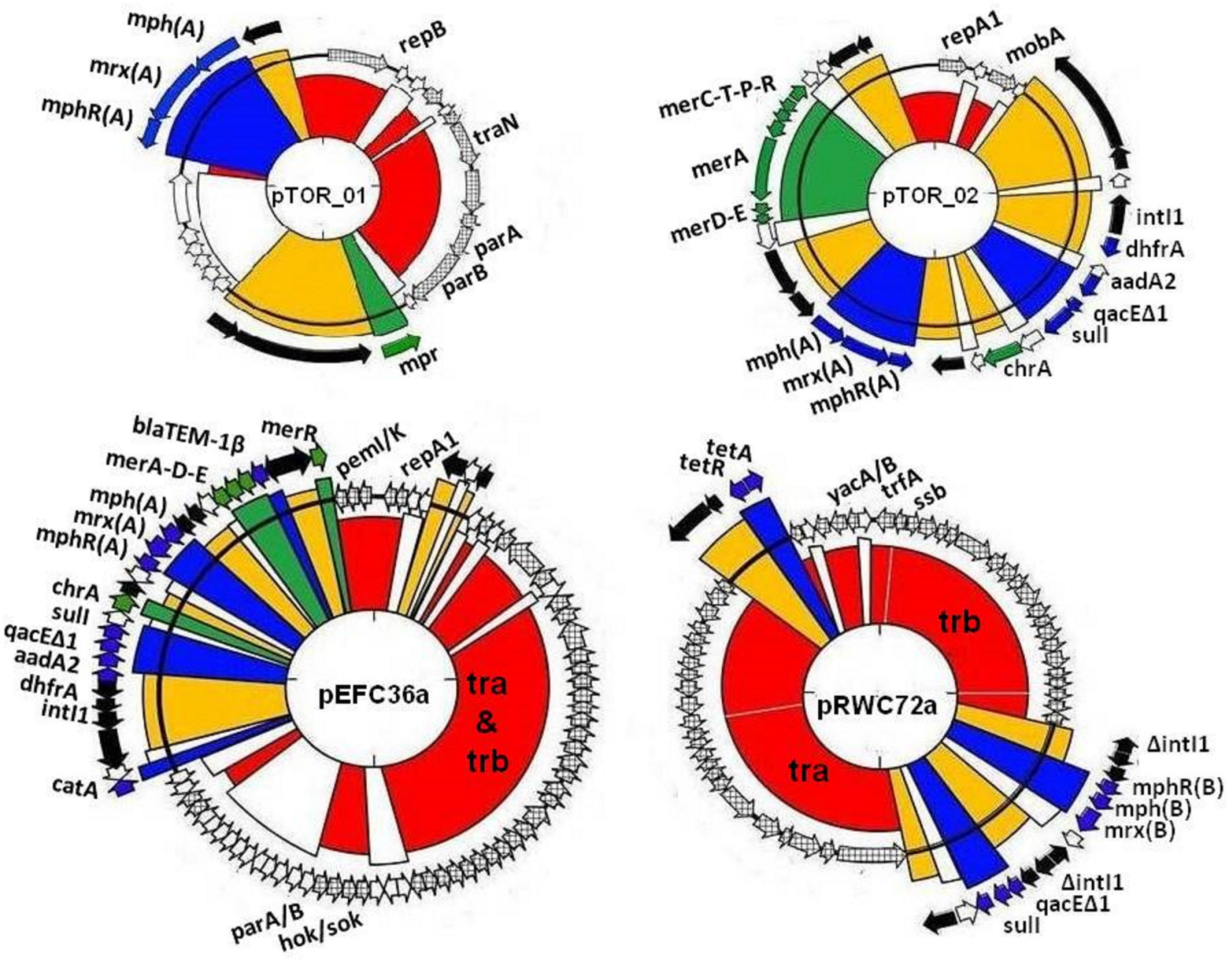
**Visual maps of multiple resistance plasmids; pTOR_01 (20,914 bp), pTOR_02 (28,080 bp), pEFC36a (87,419 bp), and pRWC72a (61,919 bp) showing mosaic features of resistance genes inserted in plasmid genetic backbones**. The different colors represent regions encoding putative functions such as replication and maintenance (red, crosshatched), antibiotic resistance (blue), heavy metal resistance (green), transposons and insertion sequences (yellow), hypothetical open read frames (white).

**Figure 2:**
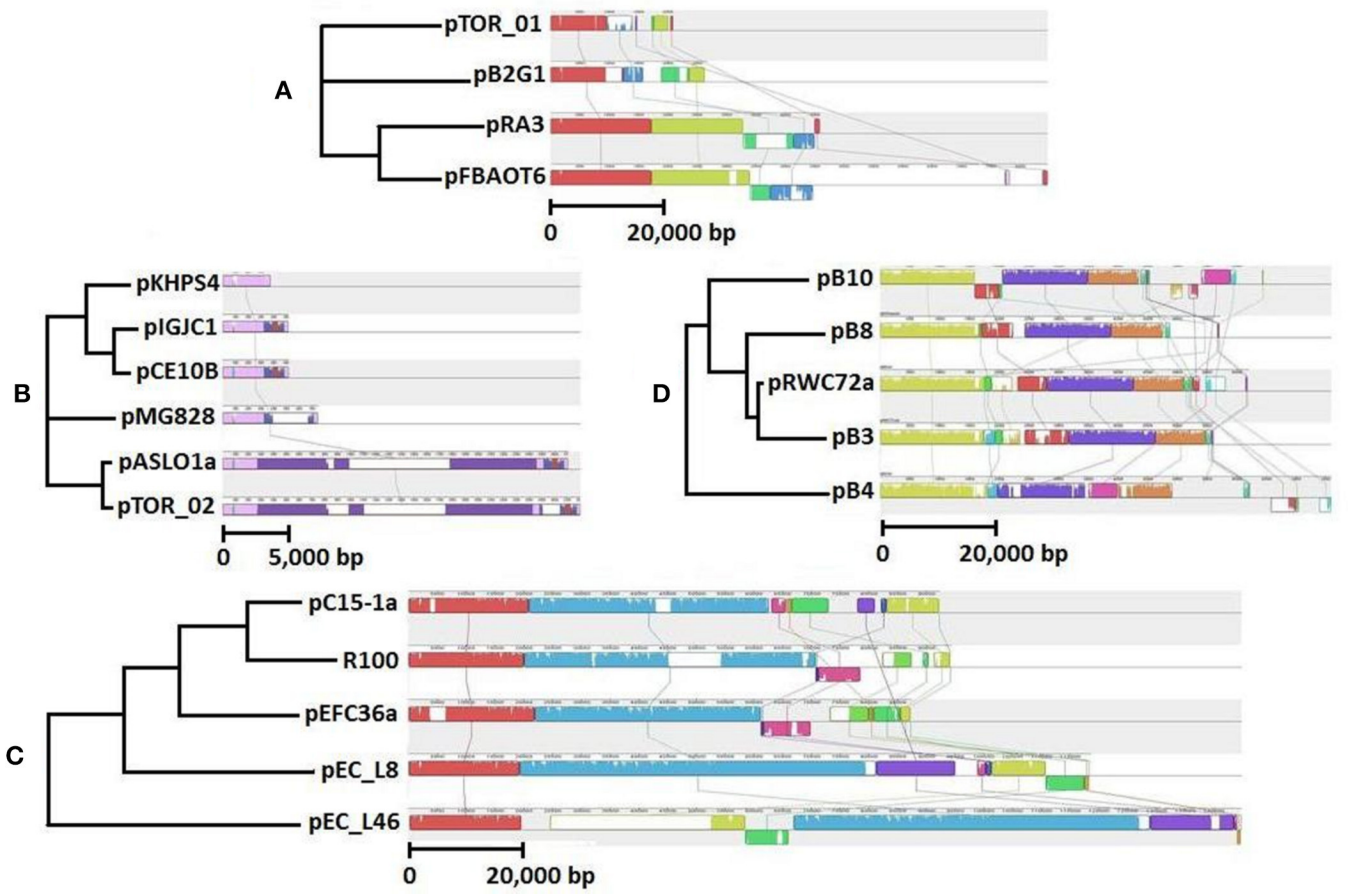
**Comparative analysis of (A) pTOR_01 and (B) pTOR_02, (C) pEFC36a and (D) pRWC72a by mauve alignment, showing evolutionary relationships with related plasmids, Genbank accession numbers are in brackets**. Same color represents regions of highest similarity. The selected sequences are closely related *Inc*U plasmids pFBAOT6 (CR376602), pRA3 (DQ401103), pP2G1 (HE616910), *col-E* related plasmids pKHPS4 (CP003226), pIGJC156 (EU090225), pCE10B (CP003036), pMG828 (DQ995354), pASL01a (JQ480155), *Inc*P-1β plasmids pB3 (AJ639924), pB4 (AJ431260) pB8 (AJ863570), pB10 (AJ564903) and *Inc*FII plasmids, R100 (AP000342), pC15-1a (AY458016), pEC_L8 (GU371928), pEC_46 (GU371929).

We screened our library of erythromycin resistance plasmids to identify ones that conferred different multiple antibiotic resistance profiles to the *E. coli* host. We also screened the plasmid collection based on plasmid size using Eckhardt gels. We used this strategy to increase our chances for identifying distinct plasmids, therefore providing a greater opportunity to characterize a diverse set of plasmids. Ultimately, we selected two larger plasmids and two smaller plasmids with different resistance profiles for DNA sequencing and further characterization. These plasmids are described in Table 1 and graphical representations can be found in Figure 1. Each plasmid represents a different plasmid incompatibility group, and the plasmids vary in their gene content related to transfer among hosts and host stability (Table 1). The plasmids share similarity to previously identified plasmids (Figure 2) and include plasmids isolated from other WWTPs and other aquatic environments and, in some cases, plasmids of clinical origin. For example, plasmid pEFC36a has high identity to sequenced *Inc*FII plasmids R100 (94,281 bp) and pC15-1a (92,353 bp); plasmid R100 was originally isolated from a clinical isolate of *Shigella flexneri* (McIntire and Dempsey, 1987), and pC15-1a was previously linked to a multi-drug resistant *E. coli* outbreak strain in Canada (Boyd et al., 2004) (Figure 2C). While, pRWC72a shows high nucleotide identity to sequenced *IncP-1β* plasmids pB3 (56,167 bp), pB4 (79,370 bp), pB8 (57,198 bp), and pB10 (64,508 bp) which were all isolated from a WWTP in Germany (Schluter et al., 2003; Heuer et al., 2004) (Figure 2D).

**Table 1:** **Summary characterization and comparison of plasmid backbones and accessory genes**.

	**Genetic backbone**					**Accessory components**		
	**Replication**	**Mobility**		**Maintenance**		**Resistance genes**		
**Plasmid (source)**	**Inc group**	**Tra genes (mob genes)**	**Trb genes**	**Plasmid addiction (TA systems)**	**^†^Regulatory and stability**	**Integron**	**Antibiotic**	**Heavy metal**
pTOR_01 20,914 bp (influent)	*rep*B/*Inc*U	N	None	None predicted	*par*A/B	None	*mph*(A), *mrx*(A), *mph*R(A)	*mpr*
pRWC72a 61,919 bp (influent)	*trf*A/*Inc*P-1β	P-T4SS; C,D,E,FGI,J,K,L,M	A,B,C,D,E,F,G,H,I,J,K,L,M,N,OP	*yac*A, *yac*B	*ssb,par*A/B, *krf*A/B,*inc*C1, *kor*B/C,*kor*A, *kle*A/B/C/E, *klc*A, *krf*A	Δ*int*I1, class1; *qac*EΔ1, *sul*I, orf5	*mph*(B), *mrx*(B), *mph*R(B), *tet*A, *tet*R	None
pTOR_02 28,080 bp (effluent)	*col*-E related	*mob*A	None	None predicted	None predicted	*int*I1, class1; *dhf*rA, *aad*A2, *qac*EΔ1, *sul*I, orf5	*mph*(A), *mrx*(A), *mph*R(A)	*mer*A, *mer*D, *mer*E, *mer*P, *mer*T, *mer*R
pEFC36a 87,419 bp (effluent)	*rep*A1/*Inc*FII	F-T4SS; B,C,D,F,G,H,IJ,K,L,M,N,S,T,U,V,W, X	A,B,C,F, I, J, O	type I TA system: *hok/sok*	conjugation: *finO, finP, traJ, traM, traY*; partition: *stb*A/B (*parM/R*)	*int*I1, class1; *dhf*rA, *aad*A2, *qac*EΔ1, *sul*I, orf5	*mph*(A), *mrx*(A), *mph*R(A), *bla*_*TEM*-1β_ *cat*A	*mer*A, *mer*D, *mer*E, *mer*R
				type II TA system: *pemI/pemK (kis/kid, mazEF);*	replication: r*epA2* (*copB*), *repA1* (*repA*), *copA*			

Inc, incompatibility; Tra, Trb, conjugative transfer modules; mob, mobilization.

† Regulatory genes involved in the control and effective functions including plasmid replication, copy number, incompatibility, and conjugative transfer. All homologs were predicted using BlastP.

Strikingly, pEFC36a shows >99% sequence identity to sequence entries available from partial sequences of plasmid R1 covering regions from *traT* to *pemI/pemK* (entries X06240, V00351, EU686388, AY423546, AY684127) as well as sequences around the *oriT* including the first genes of the transfer operon (entries X13681, M19710, X00783, X15279). Plasmid R1 was originally isolated 1962 from a patient suffering from a *Salmonella* infection in Brighton, England (Datta and Kontomichalou, 1965; Meynell and Datta, 1967). In terms of conjugation (Frost and Koraimann, 2010), plasmid replication (Nordström, 2006) and plasmid stability functions (De la Cueva-Méndez and Pimentel, 2007) it is probably one of the best characterized conjugative resistance plasmids of clinical origin.

The pTOR_01 (20,914 bp) plasmid has a highly conserved *Inc*U genetic backbone that is associated with broad host range plasmids. Plasmid pTOR_01 also carries genes homologous to a known partitioning system *par*, consisting of *par*A and *par*B. These partition genes have functions that promote distribution of plasmids to both daughter cells during host cell division (Sergueev et al., 2005). In addition, this plasmid contains a single *tra*N gene homolog but lacks other associated conjugal transfer genes and is thus predicted to be non-mobilizable. The plasmid pTOR_02 (28,080 bp) has a replication gene (*rep*A1) associated with *col*-E-related narrow host range plasmids, typically found only in *E. coli* and closely related bacteria (Riley and Wertz, 2002). Plasmid pTOR_02 has the smallest backbone carrying no predicted stability or addiction genes, which suggests that the plasmid may not be stably maintained by the host when the accessory genes it carries are not required for host survival in the environment. Plasmid pTOR_02 also lacks predicted *tra* genes but carries a single mobilization gene (*mob*A). The *mob*A gene codes for a DNA relaxase required for cleaving a specific site at the origin of replication (*ori*T) initiating plasmid DNA transfer during conjugation (Smillie et al., 2010).

The plasmid pEFC36a (87,419 bp) replication region is almost identical (>99% sequence identity) to the *Inc*FII replication region of plasmid R1 consisting of *rep*A1/repA involved in initiation of replication, and *rep*A2/copB located upstream, is a regulatory gene associated with negative regulation of replication initiation (Nordström, 2006). Genes that are identical to *parM* and *parR* (the so called *parA* locus, accession number X04268) of plasmid R1 encoding one of the best characterized active partitioning systems (Salje et al., 2010), and the *hok*/*sok* postsegregational killing genes (*parB* locus of plasmid R1, accession number X05813) are also found in the pEFC36a DNA sequence. Together with the type II antitoxin-toxin system *pemI*/*pemK* (*kis*/*kid* in plasmid R1, accession number X06240), all plasmid maintenance elements of plasmid R1 (De la Cueva-Méndez and Pimentel, 2007), are found on plasmid pEFC36a. Plasmid pRWC72a (61,919 bp) backbone is defined by a *trf*A replicon with an origin of replication (*ori*V) homologous to the incP-1β incompatibility group. This group is frequently associated with replication in a broad range of bacterial species, in fact *Inc*P-1β plasmids are often regarded as highly promiscuous particularly in the Enterobacteriaceae family (Suzuki et al., 2010). Both plasmid pEFC36a and pRWC72a have larger backbones, which consist of several putative genes that code for mechanisms responsible for plasmid inheritance and stability in bacterial hosts. Two plasmid addiction systems with homology to *hok/sok* and *pem*I/*pem*K are present on plasmid pEFC36a. Both plasmid addiction systems are common and employed by large low copy number plasmids to ensure that plasmids remain established in the bacterial population even in the absence of a selection pressure (Tschäpe, 1994). A similar addiction mechanism has been predicted in pRWC72a consisting of homologs to two genes, *yac*A and *yac*B (putative host killing toxin and anti-toxin genes). In addition to their large backbone, both plasmids carry a set of predicted *trb* and *tra* genes that are required for conjugation (Li et al., 1999; De la Cruz et al., 2010). In both cases single-stranded DNA is transferred via a type IV secretion system with pEFC36a belonging to the F-type and pRWC72a belonging to the P-type subdivisions, respectively (Bhatty et al., 2013; Bi et al., 2013).

The core backbone of pTOR_01 (9415 bp) shows 99% identity at nucleotide level to the backbones of the previously sequenced *Inc*U plasmids pFBAOT6 (84,749 bp), pRA3 (45,909 bp), pB2G1 (26,645 bp) (Figure 2A). These previously characterized plasmids were all found in *Aeromonas* species isolated from aquatic environments. Plasmid pRA3 was isolated from *Aeromonas hydrophila* in Poland, plasmid pB2G1 from a multi-drug resistant *Aeromonas* species strain isolated from a river in Spain (Marti and Balcázar, 2012), and pFBAOT6 was originally isolated from a strain of *Aeromonas caviae* from hospital sewage effluent in United Kingdom (Rhodes et al., 2000, 2004). Plasmid pTOR_01 has the highest similarity to pB2G1, as both lack a mobilization and transfer region found in plasmids pRA3 and pFBAOT6 (Figure 2A).

The plasmid pTOR_02 backbone (2751 bp) is homologous to several *col*-E plasmids including pASL01a (27,072 bp, 99%), pCE10B (5163 bp, 99%), pKPHS4 (3751 bp, 97%), pMG828 (7462 bp, 97%), and pIGJC156 (5146 bp, 95%), which were isolated from different *E. coli* strains (Figure 2B). These *col*-E related plasmids possess a small circular backbone with no predicted accessory genes that code for any resistance except for plasmids pTOR_02 and pASL01a. Plasmid pTOR_02 shows the highest similarity to pASL01a which was isolated from an *E. coli* strain found in a human fecal sample (Labar et al., 2012). Both have a derivative of Tn*21* inserted in their small and similar genetic backbone.

### Antibiotic resistance genes within the plasmids

Each plasmid carries accessory regions associated with mobile genetic elements and resistance genes, such as: class 1 integrons, antibiotic and heavy metal resistance, and transposable elements (Table 1, Figure 1). There are also several hypothetical genes, but no putative degradative or virulence associated genes were identified in the plasmid sequences. All four plasmids encode a macrolide resistance gene cluster comprised of genes that code for a macrolide 2′ phosphotransferase (*mph*), hydrophobic protein (*mrx*) and a transcriptional regulator (*mph*R). These genes are collectively involved in a phenotype of high-level resistance to erythromycin and other macrolides (Roberts, 2004). Plasmid pTOR_01, pTOR_02, and pEFC36a carry a similar macrolide resistance gene cluster designated as *mph*(A), *mrx*(A), *mph*R(A) in that order, and it has been described in previously sequenced plasmids (Noguchi et al., 1995; Poole et al., 2006; Szczepanowski et al., 2009). Alternatively, plasmid pRWC72a contains a variant form designated as *mph*R(B), *mph*(B), *mrx*(B). This macrolide gene cluster has only been described in a single multiple resistance plasmid pRSB111 (GenBank: AM260957) isolated from a WWTP in Germany (Szczepanowski et al., 2007).

In addition to macrolide resistance, plasmids pTOR_02, pEFC36a, and pRWC72a carry a class 1 integron element within their backbones that is also associated with transposable elements belonging to the Tn*21/* Tn*3* family. Tn*21* transposons frequently consist of multiple genes including the class 1 integron, and antibiotic and heavy metal resistance genes. The class 1 integron element is comprised of the 5′ conserved *int*I1 integrase gene and a 3′ conserved segment of genes; *qac*EΔ1, *sul*1, and orf5 for quaternary ammonium compounds, sulphonamide and putative puromycin resistance, respectively (Bennett, 2008; Gillings et al., 2008). The *int*I1 integrase gene is involved in excision and integration of resistance genes in cassettes located between the 5′ and 3′ conserved segments by a recombination mechanism (Zhang et al., 2009). In pRWC72a the class 1 integrase is disrupted by insertion of the macrolide (B)-resistance gene cluster, suggesting that integration of additional resistance genes within the integron gene cassettes by recombination may not be possible in pRWC72a due to the disruption in the *int*I1 gene. The pTOR_02 and pEFC36a has additional genes, *dhf*R (trimethroprim resistance) and *aad*A2 (aminoglycosides resistance) inserted as integron associated gene cassettes. Plasmid pEFC36a also carries the *bla*TEM gene, coding for beta-lactam resistance within the Tn*21* transposon, the *cat*A gene (coding for chloramphenicol resistance) is also found upstream of the Tn*21/*class 1 integron element. Plasmid pRWC72a also carries homologs of *tet*A and *tet*R genes involved in resistance to tetracyclines. Genes coding for putative resistance to heavy metals are present in plasmid pTOR_01, pTOR_02, and pEFC36a. Homologs of the *mpr* gene in pTOR_01 are associated with resistance to zinc (Picão et al., 2008). Predicted genes coding for chromium (*chr*A) and mercury (*mer*A, C, D, E,T, P, R) resistance are also found in the Tn*21* region of plasmid pTOR_02 and pEFC36a.

### Mobilization and stability of the plasmids

Both pEFC36a and pRWC72a were confirmed by conjugation experiments to have functional conjugative self-transfer modules, and both plasmids pEFC36a and pRWC72a transferred from DH5α *E. coli* host bacterium to a recipient *E. coli* at high frequencies of 4.4 × 10^−1^ and 9.7 × 10^−2^ transconjugants per donor cell. Transconjugants were screened for their acquisition of the respective antibiotic resistance phenotypes associated with each plasmid. Furthermore, these plasmids were capable of inter-species transfer and were mobilized from *E. coli* to *P. agglomerans*, albeit at lower frequencies of 2.4 × 10^−5^ and 3.6 × 10^−6^, respectively. The frequency of transfer increased for both plasmids when they transferred between *P. agglomerans* and *P. ananatis* resulting in higher frequencies of 1.6 × 10^−3^ and 3.7 × 10^−4^ for pEFC36a and pRWC72a, respectively. Both *P. agglomerans and P. ananatis* are ubiquitous environmental bacteria that can be isolated from plants, soil and water (Delétoile et al., 2009) and could represent a reservoir for resistance plasmids. They are members of the Enterobacteriaceae but, they are distantly related to other Enterobacteriaceae like *E. coli* and *Salmonella*. Therefore, successful conjugation to *Pantoea* spp. indicated that these plasmids are capable of transfer among distantly related Enterobacteriaceae members.

Furthermore, pEFC36a::Kan, the derivative of pEFC36a lacking the resistance determinant region from *merR* to *catA* (see Materials and Methods), was tested for its ability to be activated for self-transfer from an *E. coli* MC4100 donor strain in the presence of a compatible plasmid expressing the activator of the DNA transfer genes, *traJ* from plasmid R1, *in trans*. *E. coli* transconjugants (transfer frequency 1.0 × 10^−1^) were readily detected in the presence of plasmid pJR1 expressing TraJ whereas no transconjugants appeared in the vector control (transfer frequency < 1.0 × 10^−3^) indicating that TraJ of plasmid R1 could activate the P_Y_ promoter, transfer gene expression and conjugation of pEFC36a::Kan similarly to that of pAR183, a derivative of plasmid R1 (Wagner et al., 2013).

Plasmid pTOR_2 could not be mobilized from a DH5α *E. coli* host, however it was successfully mobilized from S17-1 *E. coli* (a plasmid mobilizing strain that has the pRP4 plasmid *tra* region integrated into its chromosome) to both *E. coli* DH5α and *P. agglomerans* with transfer frequencies of 3.9 × 10^−5^ and 1.5 × 10^−7^ transconjugants/donor cell, respectively. These results confirm pTOR_02 carries a functional mobilization region but requires a helper plasmid for successful conjugation. Conversely, pTOR_01 could not be transferred between *E. coli* cells in lab conjugation experiments (data not shown), confirming the prediction from the DNA sequence data that pTOR_01 is a non-mobile plasmid.

The stability of all four plasmids in *E. coli* and *P. agglomerans* grown in the absence of antibiotic selection was consistent with the predictions from the bioinformatics analysis. The plasmid stability results support the hypothesis that plasmids pTOR_01, pEFC36a, and pRWC72a have effective plasmid maintenance genes. There was no observed reduction in plasmid containing cells during 26 days of serial culturing (approximately 1200 generations). The pTOR_02 stability in both *E. coli* and *P. agglomerans* showed significant reduction in plasmid containing cells during extended culturing, and notably the pTOR_02 DNA sequence annotation did not identify any predicted plasmid maintenance genes.

## Discussion

The erythromycin enrichment approach used for trapping plasmids from environmental samples appropriately isolated plasmids harboring a macrolide resistance gene cluster. Macrolides such as erythromycin have a long history of human clinical use and associated bacterial resistance is also well documented. Erythromycin residues have been detected in Wascana Creek downstream from the WWTP (Waiser et al., 2011). Furthermore, a significantly higher concentration of erythromycin (approximately 10-fold) was observed directly downstream of the WWTP compared to an upstream sampling site (*P* < 0.0001) (Yost, 2010). Our genotypic and phenotypic characterization of these plasmids demonstrated that they also contain resistance genes for other classes of antibiotics including clinically relevant classes such as beta-lactams, chloramphenicol and tetracycline. Comparative analysis of pRWC72a may provide insights on the acquisition of multiple resistance genes in WWTP environments. Based on comparative DNA analysis, pRWC72a and the wastewater associated plasmid pB3 isolated in Germany may share a common ancestry (Figure 2D). However, the insertion of the macrolide B-resistance gene in the pRWC72a backbone is notable, because this acquisition resulted in additional resistance to erythromycin, which is not encoded by the related plasmids pB3, pB4, pB8, and pB10 (Schluter et al., 2003; Tauch et al., 2003; Heuer et al., 2004). Determining the influence of anthropogenic activities on the rates of resistance gene acquisition in plasmids from different environmental contexts will be an area of future investigation.

Plasmid pEFC36a shares high homology to clinical *Inc*FII plasmids R100, pC15_1a, pEC_L8, and pEC_L46 (Figure 2). Moreover, there is a striking conservation in DNA sequence and gene order of different key elements (plasmid replication, stability and type IV secretion genes) in comparison to plasmid R1 which was isolated in 1962 from a patient in England suffering from a *Salmonella* infection (Datta and Kontomichalou, 1965). It is very likely that plasmids R1 and pEFC36a share a common ancestor, and it is even possible that plasmid R1 is directly ancestral to pEFC36a. In addition, pEFC36a retained its ability to conjugate and conjugation could be stimulated approximately 100-fold by TraJ of plasmid R1, demonstrating functional conservation of a key regulatory feature of F-like conjugation modules (Frost and Koraimann, 2010). Thus, pEFC36a represents an example of a highly successful conjugative element that has been maintained and evolved within bacterial hosts under different environmental conditions. The presence of pEFC36a in a WWTP in Canada supports the notion that F-like conjugative elements and resistance genes integrated onto their backbone can efficiently spread and persist in bacterial populations even without continuous selective pressure (Koraimann and Wagner, 2014). A remaining challenge is quantifying the probability and transfer rate of a plasmid such as pEFC36a leaving an environmental reservior and entering a human reservoir. In this context, the fact that the water impacted by the WWTP effluent is used for irrigation of fresh produce (Fremaux et al., 2009; Tambalo et al., 2012) further emphasizes the importance of quantifying the movement of ARPs following the release from WWTPs.

The city of Regina WWTP effluent has been demonstrated to release antibiotic residues into Wascana Creek (Yost, 2010; Waiser et al., 2011). The persistence of antibiotic residues even at low concentrations has been shown to promote plasmid maintenance and horizontal mobility of plasmids among different bacterial populations in the environment (Knapp et al., 2008; Storteboom et al., 2010). Plasmid stability requires successful plasmid replication but stability in a population may also be enhanced by plasmid addiction systems. Given the stability of the plasmids pTOR_01, pEFC36a, and pRWC72a was equally high in both *E. coli* and *P. agglomerans* during lab culturing without selection of the antibiotic resistance coded on the plasmids and the presence of predicted addiction systems, it is likely that these plasmids could persist in the environment without continued antibiotic selection. Future studies are warranted to identify biotic and abiotic mechanisms in WWTPs that influence antibiotic resistance plasmid diversity in WWTP bacterial populations, their release into the environment and their subsequent fate.

### Conflict of interest statement

The authors declare that the research was conducted in the absence of any commercial or financial relationships that could be construed as a potential conflict of interest.
