# Supplementary material for: Characterization and comparative analysis of antibiotic resistance plasmids isolated from a wastewater treatment plant
Source: Front Microbiol. 2014 Oct 28;5:558. doi: 10.3389/fmicb.2014.00558 (PMC4211555; doi:10.3389/fmicb.2014.00558)
Supplement: Supplementary file 1 [file Table1.DOC]

**Supporting Table:**

Table S1. Primers used for construction of pEFC36a::Kan.

| **Name** | **Target gene/ (description)** | **Sequence 5’🡪3’** | **Location† (plasmid)** | **Amplicon size bp** | **Reference** |
| --- | --- | --- | --- | --- | --- |
| Kan_fw  Kan_rv | Kan^R^ gene | CAAGGGGTGTTATGAGCCATATTC  CTTAGAAAAACTCATCGAGCATCAAATG | pET28a | 828 | This study |
| Cat_pEFC36a_fw  Cat_pEFC36a_rv_Kan | *catA* homology + Kan extension | GCCCTGGGCCAACTTTTGGCG  gaatatggctcataacaccccttgGCCATTGGGATATATCAACGGTGG | 59796-59980 (pEFC36a) | 185+24 (extension) | This study |
| Mer_pEFC36a_fw_Kan  Mer_pEFC36a_rv | *merR* homology + Kan extension | catttgatgctcgatgagtttttctaagGGGAACCGGACAAGCCTTAC  GTGACGCGATCAACGGGCAG | 85114-85407  (pEFC36a) | 294+28 (extension) | This study |
| out_Cat_fw  out_Mer_rv | primers for confirmation of Kan^R^ insertion | GGTGTCCCTGTTGATACCGG  GGACGAGTGGGAATCCATGG | 59773-59792  85516-85535  (pEFC36a) | NA | This study |
